# Two New Triterpenoids from *Lysimachia* *heterogenea* Klatt and Evaluation of Their Cytotoxicity

**DOI:** 10.3390/molecules16098076

**Published:** 2011-09-20

**Authors:** Xin-An Huang, Xiao-Ling Shen, Ying-Jie Hu, Ya-Ming Liu, Kang-Lun Liu, Feng-Xue Zhang, Xin-Xin Zhou

**Affiliations:** 1 Tropical Medicine Institute, Guangzhou University of Chinese Medicine, Guangzhou 510405, China; Email: xahuang@163.net (X.-A.H.); xlshen66@hotmail.com (X.-L.S.); yjhu01@126.com (Y.-J.H.); LSDXLL@163.com (K.-L.L.); 2 The First Affiliated Hospital, Guangzhou University of Chinese Medicine, Guangzhou 510405, China; Email: yaminliu@yahoo.com; 3 College of Vocational and Technical Education, Guangzhou University of Chinese Medicine, Guangzhou 510405, China

**Keywords:** *Lysimachia heterogenea* Klatt, triterpenoids, cytotoxicity

## Abstract

Two new 13,28-epoxy oleanane-type triterpenoids, namely heterogenoside E and F, were isolated from *Lysimachia heterogenea* Klatt, together with the eight known compounds: palmitic acid, *β*-stigmasterol, kaempferol, quercetin, hyperin, isorhamnetin, isorhamnetin-3-*O*-galactopyranoside and anagallisin C. Heterogenoside F possesses acetoxyl groups at the unusual C-21 and C-22 positions of its oleanane skeleton. The cytotoxic activities of anagallisin C, heterogenoside E and F were weak.

## 1. Introduction

In a previous cytotoxicity-guided phytochemical screening, we found four 12-oleanene derivatives in the cytotoxic fractions of *Lysimachia heterogenea* Klatt [1]. *L . heterogenea* Klatt belongs to the genus *Lysimachia*, which is rich in triterpenoids [2]. As a continuation of our exploration of the ingredients in other fractions of this species, we report in this paper the isolation and structuralidentification of two new 13,28-epoxy oleanane-type triterpenoids, heterogenoside E (**8**) and F (**9**), isolated together with eight known compounds [palmitic acid (**1**), *β*-stigmasterol (**2**), isorhamnetin (**3**), kaempferol (**4**), quercetin (**5**), isorhamnetin-3-*O*-galactopyranoside (**6**), hyperin (**7**) and anagallisin C (**10**)]. The cytotoxic activities of compounds **8**, **9** and **10** were also evaluated.

## 2. Results and Discussion

### 2.1. Structure Elucidation of New Compounds

*Heterogenoside E* (**8**), obtained as white powder (MeOH), had the molecular formula of C_46_H_74_O_17_ according to its HRESIMS data. The degrees of molecular unsaturation were calculated to be 10, among which three sugar rings, deduced from the reaction products of the chemical analysis and three pairs of anomeric protons and carbons in ^1^H- and ^13^C-NMR spectra (Table 1), accounted for three degrees, and the C=O group inferred from the *δ* 212.0 peak in the ^13^C-NMR spectrum was another one.

**Table 1:** ^1^H- and ^13^C-NMR data for the sugar moieties of compounds **8** and **9**.

No.	Compound 8		Compound 9	
	C	H	C	H
arabinose'				
1	106.5	4.87 (*d*, 7.5 Hz)	104.6	4.78 (*d*, 6.0 Hz)
2	81.1	4.08 (*brs*)	79.7	4.57 ^a^
3	73.9	4.25 ^a^	73.2	4.30 ^a^
4	74.5	4.31 ^a^	78.4	4.31 ^a^
5	66.5	3.65 (*d*, 12.5 Hz), 4.60 (*d*, 12.5 Hz)	64.1	3.68 (*m*), 4.65 (*dd*, 12.0 Hz, 4 Hz)
glucose'' (at C-2 of arabinose)				
1	105.3	5.01 (*d*, 7.5 Hz)	104.9	5.50 (*d*, 7.5 Hz)
2	86.2	3.95 ^a^	76.0	3.70-4.20 ^a^
3	77.9	3.80-4.30 ^a^	77.8	3.80-4.30 ^a^
4	71.0	4.25 ^a^	71.9	4.25 ^a^
5	78.2	3.80-4.30 ^a^	78.2	3.80-4.30 ^a^
6	62.4	4.32 (*m*), 4.45 (*m*)	63.0	4.60 (*m*)
glucose''' (at C-4 of arabinose)				
1			104.1	5.02 (*d*, 7.5 Hz)
2			85.4	3.91 (*m*)
3			77.6	3.80-4.30 ^a^
4			71.1	4.21 ^a^
5			77.9	3.80-4.30 ^a^
6			62.4	4.32^ a^
xylose (xylose''' for **8** and xylose'''' for **9**)				
1	108.0	4.89 (*d*, 6.5 Hz)	107.6	4.93 (*d*, 6.0 Hz)
2	76.2	3.80-4.30 ^a^	76.2	3.70-4.20 ^a^
3	77.6	3.80-4.30 ^a^	78.3	3.80-4.30 ^a^
4	70.4	3.80-4.30 ^a^	70.7	4.15 ^a^
5	67.2	3.48 (*m*), 4.28 ^a^	67.4	3.72 (*m*), 4.56 ^a^

^a^ The signals were overlapped.

The remaining six degrees, six distinct quaternary methyl groups, and the saturated carbons in high field of the ^1^^3^C-NMR spectrum all implied the existence of a saponin aglycon (Table 2).

**Table 2:** The main ^1^H and ^13^C-NMR data for the aglycon moieties of compounds **8** and **9** (125 MHz in pyridine-*d*_5_).

No.	Compound 8		Compound 9	
	C	H	C	H
1	39.1	1.02 ^a^, 1.72 (*m*)	39.2	0.85 ^a^, 1.63 (*m*)
2	26.1	2.02 (*m*), 2.23(*m*)	26.5	1.90 (*m*)
3	81.9	4.24 ^a^	88.9	3.12 (*dd*, 11.5 Hz, 4.0 Hz)
4	43.6		39.7	
5	47.4	1.58(*m*)	55.6	0.63 (*d*, 11.5 Hz)
6	17.5	1.66 (*m*)	17.8	1.40 ^a^
7	33.6	1.04 ^a^, 1.47 ^a^	34.2	1.32 ^a^
8	43.0		42.6	
9	50.3	1.28 (*m*)	50.4	1.23 ^a^
10	36.7		36.8	
11	18.9	1.26 ^a^, 1.51 ^a^	19.1	1.26-1.51 ^a^
12	31.7	1.52 (*m*)	32.6	1.52 (*m*)
13	86.2		86.0	
14	49.8		44.7	
15	45.8	1.91 (*d*, 15 Hz), 2.82 (*d*, 15 Hz)	32.8	1.45 (*m*)
16	212.0		78.4	3.80-4.30 ^a^
17	56.1		50.9	
18	54.6	2.01 (*m*)	49.4	1.85 (*m*)
19	40.0	1.40 (*m*)	38.3	1.46 ^a^, 2.64 (*t*, 15.0 Hz)
20	31.8		37.1	
21	35.6	1.19 (*m*), 1.79 (*m*)	80.4	5.80 (*d,* 10.0 Hz)
22	25.0	2.24 ^a^	74.3	4.30 ^a^
23	64.3	3.69 ^a^, 4.33 ^a^	28.0	1.23 ^a^
24	13.3	0.96 (*s*)	16.6	1.08 (*s*)
25	16.7	0.94 (*s*)	16.3	0.81 (*s*)
26	18.8	1.32 (*s*)	18.3	1.25 ^a^
27	21.7	1.01 (*s*)	19.8	1.27 ^a^
28	75.1	3.50 (*m*)	76.2	4.10 ^a^
29	33.3	0.86 (*s*)	30.3	1.13 (*s*)
30	23.5	0.81 (*s*)	20.2	1.10 (*s*)
AcO (at C-21 of aglycon)				
CO			171.1	
Me			21.0	2.00
AcO (at C-22 of aglycon)				
CO			169.7	
Me			21.9	2.40

^a^ The signals were overlapped.

**Figure 1:**
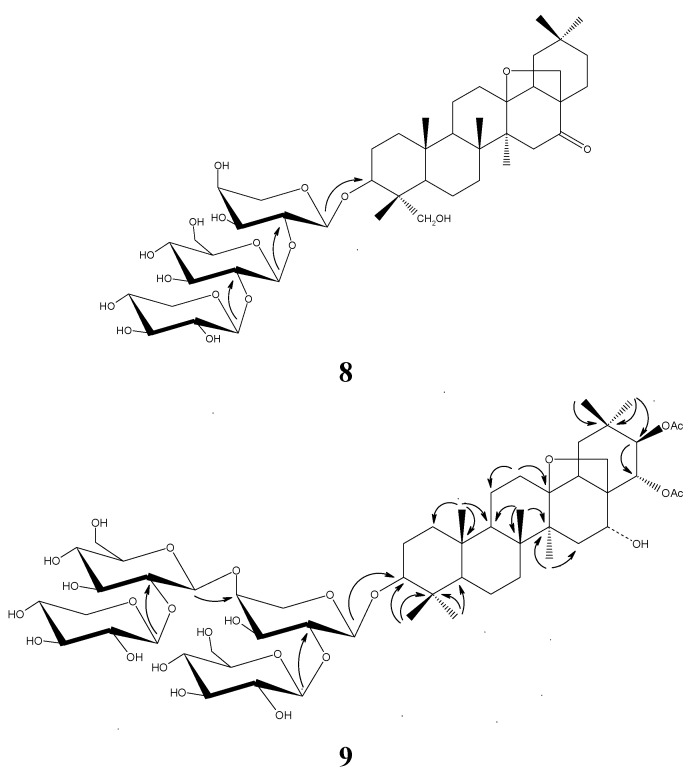
The structures and key correlations for compounds **8** and **9**.

The HMBC correlations from H-24 to C-3, C-4, C-5 and C-23; H-25 to C-1, C-9 and C-10; H-26 to C-7, C-8 and C-14; H-27 to C-8 and C-14; H-29 to C-19, C-20, C-21 and C-30; H-30 to C-20, C-21 and C-29; and H-18 to C-13; as well as the consistency of carbon signals with those reported in [3], confirmed the aglycon to be anagalligenone. The carbon chemical shifts of the sugar moieties were identical to those of heterogenoside B as reported in reference [1], and the HMBC correlations from H'-1 to C-3, H''-1 to C'-2 and H'''-1 to C''-2, and the coupling constants of the anomeric protons at *δ* 4.87 (*J* = 7.5 Hz), 5.01 (*J* = 7.5 Hz) and 4.89 (*J* = 6.5 Hz) determined the glycosyl linkage as 3-*O*-{*β*-*D*-xylopyranosyl-(1→2)-*β*-*D*-glucopyranosyl-(1→2)-*α*-*L*-arabinopyranosyl}. Therefore, it was concluded that **8** was anagalligenone 3-*O*-{*β*-*D*-xylopyranosyl-(1→2)-*β*-*D*-glucopyranosyl-(1→2)-*α*-*L*-arabinopyranosyl} (Figure 1).

*Heterogenoside F* (**9**), white powder (MeOH), had the formula C_56_H_90_O_25_ according to its HRESIMS data, and the DEPT and HMBC spectra revealed two AcO groups (Table 2). Four pairs of anomeric protons and carbons in ^1^H- and ^13^C-NMR spectra (Table 1), the carbon profile similar to that of heterogenoside D, the coupling constants of the anomeric protons at *δ* 4.78 (*J* = 6.0 Hz), 5.50 (*J* = 7.5 Hz), 5.02 (*J* = 7.5 Hz) and 4.93 (*J* = 6.0 Hz), and the HMBC correlations from H''-1 to C'-2, H'''-1 to C'-4, H''''-1 to C'''-2 supported the existence of a *β*-*D*-xylopyranosyl-(1→2)-*β*-*D*-glucopyranosyl-(1→4)-[*β*-*D*-glucopyranosyl-(1→2)]-*α*-*L*-arabinopyranosyl moiety. The remaining eight degrees of unsaturation were assigned to the AcO moieties and a six-ring triterpenoid aglycon. HMBC correlations from H-21 to carbon at *δ* 171.1 and C-22; H-23 to C-3, C-4, C-5, C-24; H-24 to C-3, C-4, C-5 and C-23; H-25 to C-1, C-5, C-9 and C-10; H-26 to C-7, C-8, C-9 and C-14; H-27 to C-8, C-13, C-14 and C-15; H-29 to C-19, C-20, C-21 and C-30; H-30 to C-20, C-21 and C-29; led to the construction of 13,28-epoxy-3,16,21,22-tetrol oleanane. Extensive analysis of the correlation of H-29 and H-21 in the NOESY spectrum, the coupling constant of H-21 (*J* **=** 10.0 Hz) and the referenced structures in the literature [4,5] implied the *α* and *β* configuration of H-21 and H-22, respectively. The HMBC correlation from H'-1 to C-3 indicated that C-3 was linked to the glycon, thus, **9** was finally elucidated as 21,21-*di*-*O*-acetyl-13,28-epoxy-3*β*,16*α*,21*β*,22*α*-oleananetetrol 3-*O*-{*β*-*D*-xylopyranosyl-(1→2)-*β*-*D*-glucopyranosyl-(1→4)-[*β*-*D*-glucopyranosyl-(1→2)]-*α*-*L*-arabinopyranosyl } (Figure 1).

The structures of the other isolated components palmitic acid (**1**), *β*-stigmasterol (**2**), isorhamnetin (**3**), kaempferol (**4**), quercetin (**5**), isorhamnetin-3-*O*-galactopyranoside (**6**), hyperin (**7**) and anagallisin C (**10**) were determined by comparison to the ^1^H and ^13^C-NMR spectral data in the literature [6,7,8,9,10].

### 3.2. The Cytotoxic Activity of Anagallisin C, Heterogenoside E and F

The biological assay results showed that the IC_50_ values of anagallisin C against the Hela, KB-3-1 and HepG_2_ cells were 35.7 ± 6.0, 30.7 ± 2.9 and 54.4 ± 5.4 μM, while the corresponding values of heterogenoside E were 31.4 ± 3.9, 34.0 ± 3.9 and 31.7 ± 4.9 μM, and those of heterogenoside F were 12.7 ± 1.2, 23.2 ± 9.6 and 21.0 ± 3.7 μM, respectively.

## 3. Experimental

### 3.1. General

The experimental instruments for structural identification, the collection of the plant *Lysimachia heterogenea* Klatt, the preparation of fractions by liquid partition and column chromatography, and the methods of chemical analysis were as previously described [1,11].

### 3.2. Extraction and Isolation

The petroleum ether, EtOAc and LH-2 fractions from *L. heterogenea* were collected as previously described [1], and every fraction was further chromatographed. The petroleum ether fraction (30 g) was subjected to silica gel column eluting with petroleum ether–acetone (95:5 and 85:15, v/v) to give compounds **1** (200 mg) and **2** (500 mg). The EtOAc fraction (40 g) was chromatographed over silica gel with MeOH-CHCl_3_ (10:90 and 20:80, v/v) to afford compounds **3** (40 mg), **4** (12 mg), **5** (60 mg), **6** (9 mg), and **7** (7 mg). The LH-2 (20 g) fraction was further purified by silica gel chromatography with MeOH-CHCl_3_ (20:80 and 25:75, v/v) to yield compounds **8** (20 mg), **9** (15 mg), and **10** (8 mg).

### 3.3. Compound Characterization

*Heterogenoside* *E* (**8**): mp 232–234 °C; −20.0 (*c* 0.25, MeOH); IR (KBr); *v* 3392, 1076 and 1044 (OH), 2946 (CH_3_), 2924 (CH_2_), and 1704 (16-ketone) cm^−1^; ^1^H- and ^13^C-NMR spectral data were listed in Table 1 and Table 2; HRESIMS *m/z*: 897.4853 [M-H]^−^ (calcd 897.4848).

*Heterogenoside* *F* (**9**): mp 216–217 °C; −24.9 (*c* 0.28, MeOH); IR (KBr); *v* 3396, 1077 and 1043 (OH), 2922 (*br*. CH_3_, CH_2_), and 1721 (ester) cm^−1^; ^1^H- and ^13^C-NMR spectral data were listed in Table 1 and Table 2; HRESIMS *m/z*: 1161.5701 [M-H]^−^ (calcd 1161.5693).

### 3.4. Cytotoxicity Bioassays

The human epidermoid carcinoma cell line KB-3-1, human hepatocellular liver carcinoma cell line HepG_2_, and human epithelial carcinoma cell line Hela cells (5 × 10^3^ cells/well) were cultured in 96-well plates for 24 h, respectively, then a buffer solution (100 μL) containing the test compounds at various concentrations (100, 25, 6.25, 1.25 μg/mL) was added to each well. The cells, treated with those compounds and DMSO (as the control), were incubated for 72 h at 37 °C in a humidified chamber. The culture was terminated by adding MTT solution (20 μL, 5 mg/mL) to each well, and a further incubation for 4 h was performed. After the suspension was removed from each well, DMSO (100 μL) was added and mixed thoroughly. The absorbance was measured by a microplate reader at 492 nm with 620 nm as reference, and mean IC_50_ values were calculated.

## 4. Conclusions

Eight known compounds (palmitic acid, *β*-stigmasterol, kaempferol, quercetin, hyperin, isorhamnetin, isorhamnetin-3-*O*-galactopyranoside and anagallisin C), as well as two new triterpenoids, named heterogenoside E and F, were isolated from *Lysimachia heterogenea* Klatt. Heterogenoside F is a rare 21,22-diacetoxyl triterpenoid. The cytotoxic activities of anagallisin C, heterogenoside E and F were weak.
